# Indoor Air Pollution in Cars: An Update on Novel Insights

**DOI:** 10.3390/ijerph16132441

**Published:** 2019-07-09

**Authors:** Nicole Zulauf, Janis Dröge, Doris Klingelhöfer, Markus Braun, Gerhard M. Oremek, David A. Groneberg

**Affiliations:** Institute of Occupational, Social and Environmental Medicine, Goethe-University, 60590 Frankfurt, Germany

**Keywords:** car indoor, air pollution, volatile organic compounds (VOC), carbon oxides (COx), particulate matter (PM), airborne bacteria, fungi, (novel) brominated flame retardants ((N)BFR), organophosphate flame retardants (OFR), high molecular weight plasticizer, nitrogen oxides (NOx)

## Abstract

From a global viewpoint, a lot of time is spent within the indoor air compartment of vehicles. A German study on mobility has revealed that, on average, people spend 45 minutes per day inside vehicles. In recent years the number of cars has increased to around 43 million vehicles in private households. This means that more than one car can be used in every household. The ratio has been growing, especially in eastern Germany and rural areas. “Overall and especially outside the cities, the car remains by far number one mode of transport, especially in terms of mileage”. Therefore, numerous international studies have addressed different aspects of indoor air hygiene, in the past years. In this paper, meaningful original studies on car indoor air pollution, related to VOCs, COx, PMs, microbials, BFRs, OPFRs, cigarettes, electronic smoking devices, high molecular weight plasticizer, and NOx are summarized in the form of a review. This present review aimed to summarize recently published studies in this important field of environmental medicine and points to the need for further studies with special recommendations for optimizing the interior air hygiene.

## 1. Introduction

“Overall and especially outside the cities, the car remains by far number one mode of transport, especially in terms of mileage” [1].

“Health is a state of complete physical, mental and social well-being and not merely the absence of disease or infirmity”—definition provided by the World Health Organization (WHO) [2]. Indoor “bad air” is often a cause of discomfort, but beyond this, it can also lead to serious illnesses. Through breathing, the pollutants in indoor air also pass on into our bodies. Therefore, air quality plays a relevant role in environmental and occupational medicine and many airborne factor adversely influence human health [3,4]. This review merges recent data on in-car air quality that has been published by various research groups, all over the world. In general, air pollution refers to the release of pollutants into the air, which are harmful to the environment and health. It is the emission of toxic ingredients into the atmosphere from anthropogenic (mainly related to motorized street traffic like tire abrasion and exhaust gases) or natural sources [5]. In many parts of the world, air pollution is still dangerously high and poses a great danger to health and climate [2].

“Every year supposed 7 million people worldwide die of air pollution in cause of fine dust particulates that induces severe diseases such as increased cardiovascular risks, heart disease, stroke, lung cancer, chronic obstructive pulmonary disease (COPD), asthma and respiratory tract infections, including pneumonia. A report of the WHO suggested that 9 of 10 people breathe air with high pollutant content with the consequence of 4.2 million deaths anniversary as a consequence of exposure to surrounding air pollution“ [6].

Currently, the emission (exhaust and non-exhaust) of motor vehicles represents a heavily debated issue. Thus, this article aims to address an important aspect of air pollution—indoor air pollution in cars. This is a follow-up of a review that was published 2011 by Müller et al. [5]. Rather than summarizing the studies with general aspects of air quality, this review specifically focuses on the indoor air environment in cars.

## 2. Possible Pollutants

### 2.1. Volatile Organic Compounds (VOCs)

A recently published report of Bakhtiari et al. focused on measurements of benzene, toluene, ethylbenzene, xylene (BTEX), acetaldehyde, and formaldehyde concentrations in indoor car compartments of different models and ages, before and after the refueling process, with different fuel types (compressed natural gas (CNG), gas, and liquefied petroleum gas (LPG)). The average levels of formaldehyde ranged between 806 (+/-323) and 1,144 (+/-240) ppbv, respectively [7]. In 2016, the Committee for Indoor Objectives (AIR) derived a benchmark I (precautionary value) of 0.1 mg per cubic meter of indoor air. This corresponds to 100 micrograms/ m^3^. This value should not be exceeded even for a short term [8]. The World Health Organization (WHO) recommends a guideline of 0.08 ppm (0.1 mg/m^3^) [9]. Acetaldehyde average concentrations showed results between 410 (+/-223) and 482 (+/-91) ppbv, respectively. The BTEX reached values below the guideline amount for all used car models. Interestingly, refueling increased the in-cabin levels of pollutants primarily the CNG and LPG fuels—the concentrations of the above-mentioned BTEX were found to be significantly higher for gasoline, during each observation [7].

In 2016, VOCs of various cars were measured in a closed-cabin environment under multiple ventilation and engine scenarios. Three different idling modes were used (engine and ventilation off, exterior air ventilation with engine idling, internal air recirculation with engine idling). The levels of the 24 VOCs averaged 4.58 ± 3.62 ppb, with a range of 0.05 (isobutyl alcohol) to 38.2 ppb (formaldehyde)). Moreover, the ‘idling engine’ concentration (5.24 ± 4.07) was 1.3–5 times greater than the ‘engine off’ one (4.09 ± 3.23). Seven VOCs showed results under the detection limit, while the other seventeen were ±2 ppb of the ambient air levels. Most outdoor VOC air values were temporally constant. Interestingly, the in-cabin levels of acetone, acetaldehyde, and formaldehyde) were significantly greater than the exterior air, regardless of ventilation or engine surroundings. VOC concentrations can reverse their outgassing from the products used in the automobile construction [10].

Barnes et al. found that 24% of examined cars passed the required Indoor Air Quality (IAQ) concentration in public places and offices for VOCs, with a recommendation of 261 ppbv. For an idle engine (mean 1,351 ppbv), the VOC concentration was significantly higher than when the car is in motion (mean 331 ppbv). A significant positive correlation (0.319, *p* < 0.05) between car age and VOCs was acquired [11]. 

Another study on taxi drivers and passengers demonstrated that the health risk of in-car benzene homologues for male drivers is the highest (1.04, 6.67), being 6.94 times that of female drivers, and female and male passengers. Thus, the cancer health risk to drivers is 1.21 (1.21E-04) times more than the acceptable value (1.00E-04) provided by the United States Environmental Protection Agency. The authors also studied the in-car benzene values (X, μg/m^3^) for male (Y = 1.48E-06X) and female drivers (Y = 1.42E-06X), and male (Y = 2.22E-07X) and female passengers (Y = 2.13E-07X) [12].

In 2014, the same authors identified the following in-car levels—benzene (82.7 μg/m^3^), toluene (212.3 μg/m^3^), ethylbenzene (74.7 μg/m^3^), xylenes (182.3 μg/m^3^), styrene (24.7 μg/m^3^), butyl acetate (33.5 μg/m^3^), undecane (61.3 μg/m^3^), and total VOCs (1,441.7 μg/m^3^). Interestingly, the concentrations rise with the temperature and relative humidity and drop with the car age and total mileage. They are higher in cars with small cabins and change with different car models and various sampling locations. Chen et al. define the car age as the most important factor influencing airborne VOCs pollution, followed by in-cabin temperature and total mileage [13].

Xiong et al. derived a theoretical formula for the correlation between the cabin VOC level (Ca) and temperature (T). Here, the logarithm of Ca/T0.75 was a linear relationship with 1/T. For this purpose, chemical emissions in three car cabins were measured at different temperatures (24 °C, 29 °C, 35 °C). Hence, the cabin VOC concentrations were obtained, which could be helpful in evaluating temperature-dependent interior emissions [14].

Similarly, Yang et al. derived a theoretical relationship between the VOC emission quantity (M) and temperature (T) with a linear logarithm of MT−1/8 with 1/T, which followed a good linear association (R2: 0.89–0.99). Hence, the VOC emission at different temperatures could be forecasted [15].

In summary, the in-cabin VOC emissions are highly dependent on variations in ventilation and engine conditions [10], as well as gender, and role as a driver or a passenger [12]. Another conclusion drawn from this paper was that these were positively associated with the car’s age, and the concentrations were greater for an idle engine than during driving [11]. The age of the car contrarily influenced formaldehyde and acetaldehyde [7]. Generally, indoor VOC levels expanded as the temperature difference between interior and exterior air increased [10]. Moreover, the importance of temperature influence on the emissions of VOCs in automobiles should also be considered. For example, when vehicles are parked in the sun, the air temperature can be very high. This can greatly enhance the emissions of pollutants from source materials. The concentrations rose with temperature and humidity, whereas they dropped with the car age and total mileage [13]. Furthermore, the procedure of filling-up the tank and replacing gasoline with CNG and LPG can be ruled out as possibilities to enhance in-cabin air concentrations. The air pollutant types and in-vehicle levels can feature relevant health impacts [7]. 

### 2.2. Carbon Oxides (COx)

Excess CO_2_ concentrations, however non-toxic, could lead to depressed reactions in the driver, sleepiness, etc., and therefore, it could contribute to increased accident rates, for example. Therefore, further noxious composites containing carbon dioxide (CO_2_) might play a role in indoor air quality. It is known that during the recirculation mode of HVAC (heating, ventilation, and air conditioning) the in-cabin CO_2_ level increases due to passenger exhalation. Luangprasert et al. performed experiments to determine CO_2_ concentration during a typical commute. In both recirculating and outside air modes, the analyses conformed to the level predicted using first-order mass balance equation. Moreover, the long-term transient concentration-decay (the Fickian diffusion process) during car-parking and switching-off of the HVAC system was also investigated. The article also includes operational details of the automotive HVAC system and fresh air ventilation exchange between the cabin exterior and interior. Here, the authors concluded that the usage of HVAC recirculation mode leads to excessive composition of cabin CO_2_ concentrations. [16].

In a study by Barnes et al., 96% of the tested cars exceeded the suggested CO_2_ measurements of 1,000 ppmv, during driving (mean 3,413 ppmv); 16% of the vehicles > 5,000 ppmv. A total of 90% surpassed the recommendation during engine idling (mean 3,096 ppmv). In all cars, carbon monoxide (CO) levels were lower than the IAQ suggestion (1.7 ppmv). This was determined in 40% of the vehicles during engine idling and in 35% during driving [11].

Another study in 2018 also investigated the in-cabin accumulation of CO_2_ and the influence of driving and ventilation [17]. The results here confirmed Luangprasert et al.´s statements mentioned above [16]. By recirculating indoor air and closing windows, in-car particulate levels are lowered. Only the CO_2_ exhalation of the occupants increase, in these cases. Circumstances like number of occupants, vehicle age, ventilation setting, cabin volume, speed, and trip duration have a great influence on different CO_2_ concentrations. Only by recirculating indoor-air setting, the CO_2_ levels can exceed the threshold of interest (2,500 ppm), which causes negative cognitive or physiological consequences, such as difficulty in concentrating and fatigue). However, under this ventilation adjustment a 2,500 ppm limit would not be reached for one or two-passenger average-duration drives. For longer excursions or more commuters, the recirculation setting should be interrupted or it should be mixed with outside air so as to not surpass CO_2_ aggregations of 2,500 ppm [17]. 

Alameddine et al. developed a framework to quantify commuter exposure to CO and PM2.5. Between the exhaust temperature and CO exposure, there is a non-linear relationship. The exposure was modulated by vehicle model, street circumstances, and weather conditions. Here, the main source of in-cabin exposure was the ambient environment. Ventilation settings could influence the in-vehicle PM2.5 levels, but not the CO ones. Wind conditions had a significant impact on in-cabin CO and PM2.5 levels. Finally, street conditions were also necessary factors—highway commuting was correlated with lower CO concentrations; higher PM2.5 levels were found by low stopping frequencies [18].

Abi-Esber et al. have also investigated the in-vehicle exposure to PM2.5 and CO. They were unexpectedly higher in new cars compared to old ones. Interestingly, there also exists a correlation to the air quality of the front area of the windshield. Significant correlations could be found between indoor and outdoor pressure differences and PM2.5, CO In/Out (IO) ratios, under air recirculation and window half-opened ventilation settings (higher correlations in the case of AC Rec (22.1 and 26.7% of PM2.5 and CO IO ratio variations) compared to W1/2O (15.7 and 17.3% of PM2.5 and CO:IO ratio variations). The humidity and temperature difference influenced the CO:IO ratios, only under the air recirculation ventilation setting [19].

### 2.3. Particulate Matter (PM) (and Microbials)

In addition to the above-mentioned values obtained by Barnes et al., this study also determined particulate matter (PM0.3 and PM2.5), fungi, and airborne bacteria concentrations, during a routine travel trip of 51 cars. Generally, during driving, in-vehicle PM2.5 concentrations decreased (driving—40 ± 28 PtL^−1^, idle engine—23 ± 19 PtL^−1^), but no such effect was seen for the PM0.3 concentrations. On the contrary, microbial concentrations were relatively small. The mean bacterial count was 350 CFUm^−3^ and mean fungal count was 13 CFUm^−3^. Interestingly, a significant positive correlation (0.30, *p* < 0.05) was found between bacterial counts and PM2.5 [11]. 

Dröge et al. examined the in-car PM levels under various settings and compared it with stationary results. For PM10, the values had a peak at 508 µg/m^3^, for PM2.5 it was 133.9 µg/m^3^, and for coarse particles it was 401.3 µg/m^3^. Smaller particles had a low fluctuation, contrary to the coarse particles with a high fluctuation (maximum values on busy roads). The PM10 values were the best indicator for traffic-based PM pollution. The window-closure lowered the PM and coarse particle concentrations. The stationary detected PM values varied significantly from the mobile PM results [20].

A study with a focus on the improvement of the in-cabin air quality found out that that by using a PM2.5 filter in the main air handling system, interior PM2.5 concentrations could be lowered from 100 µg/m^3^ to less than 25 µg/m^3^, in 100 s and to 5 µg/m^3^ in 250 s [21]. 

In case there was a potential risk for cardiovascular events, a study on 60 healthy subjects could demonstrated decrease in HRV indices while showing increased levels of in-cabin PM2.5. This was associated with autonomic alteration. The authors suggested the use of air conditioning for improving air quality [22].

Furthermore, an interesting study from 2019 investigated the correlation between emissions from a leading vehicle (LV) and the in-cabin PM concentrations in the study vehicle (SV) that was immediately following it. The in-cabin PM levels were significantly influenced by the LV’s Euro emission standard. For petrol-fuel, the median was statistically lower (e.g., −34% for PM0.3-1) with strict emission standards than that with a low-emission standard; for diesel-fuel, the median showed a strong and significant decrease (up to −62%, −44%, and −48% for UFPs (ultrafine particles), PM0.3-1, and PM1-2.5) for recent-emission standards in contrast to older-emission standards. Moreover DPF (diesel particulate filter)-equipped LVs also lead to reduced PM values (UFPs—47% reduction compared to diesel-fueled (non-DPF), PM0.3-1—80% reduction compared to both petrol-fueled and diesel-fueled (non-DPF), PM1-2.5—38% reduction compared to petrol-fueled and 46% reduction compared to non-DPF [23].

### 2.4. Legacy and Novel Brominated Flame Retardants ((N)BFRs)

Additional substances that might accumulate in vehicles are brominated flame retardants (BFRs), which are organobromine ingredients with an inhibitory influence on ignition chemistry that tends to lessen the products´ flammability. Due to the restriction of using Deca-, Penta-, and Octa-BDE (Brominated Diphenyl Ether) technical assemblies, the requirement for Novel BFRs (NBFRs) as substitutes for these formulations increased. Besis et al. examined the incidence of legacy and NBFRs in in-car dust for cars aged 1 to 19 years, with different characteristics and origin. The parameter contained 20 Polybrominated Diphenyl Ethers (PBDEs) ((Di-to Deca-BDEs), 4 NBFRs like 1,2-bis(2,4,6-tribromophenoxy)ethane (BTBPE), Decabromodiphenylethane (DBDPE), 2-ethylhexyl-2,3,4,5-tetrabromobenzoate (TBB), bis(2-ethylhexyl)-3,4,5,6-tetrabromophthalate (TBPH), tetrabromobisphenol A (TBBPA), and 3 isomers of hexabromocyclododecane (HBCD)). The results of 20PBDE varied from 132 to 54,666 ng/g (median 2,888) being dominated by BDE-209. Moreover, the levels of 4NBFRs showed measurements from 48 to 7626 ng/g (median 1,188) and were henpecked by DBDPE, the major alternative of BDE-209. HBCDs reached values between <5 and 1745 ng/g (median 155), with alpha-HBCD being the most prevailing isomer. Terminally, the TBBPA-levels diversified from <10 to 1064 ng/g (median 45). The formation profiles and deliverables of BFRs were examined with reference to the types of cars (like the country of origin, year of manufacture, and interior configuration). Here, the average daily assimilation of a few BFRs (like DBDPE, BDE-47, BDE-209, BDE-99, BDE-153, BTBPE, TBB, HBCDs, TBBPA, and TBPH) via dust ingestion and dermal absorption showed that the intake by the former was higher and was generally greater for infants than for adults.

In summary, the authors found that the potential health risk with regards to the BFRs mentioned above was some dimension of magnitude lower than the reciprocal reference dose (RfD) measurements [24].

Harrad et al. also measured PBDEs, HBCDs, and TBBP-A of in-vehicle dust. As cabin dust concentrations of HBCDs, TBBP-A, and BDEs 47, 85, 99, 100, 153, 154, 183, 196, 197, 202, 203, and 206–209 exceeded significantly (p<0.05) those in the trunk dust, it seems to be a better indicator of human exposure. In five cars, levels of TBBP-A and PBDEs 154 and 206–209 were significantly higher (*p* < 0.05) in the front seats, in comparison to other seating areas. Here too, via dust ingestion the in-vehicle exposure to PBDEs, HBCDs, and TBBP-A exceeded that via inhalation [25].

Furthermore, a Czech study group investigated the concentrations of PBDEs, HBCDs, and BFRs in car interior dust. The PBDEs reached concentrations of up to 33,728 μg/kg, respectively (maximum for BDE 209). The levels of Penta-, Octa-, and DecaBDE confirmed lower contamination of dust from Europe in comparison to North America. Among the ‘alternative’ BFRs, mainly DBDPE and HBCD were measured in the concentration ranges <20–3567 and <0.3–950 μg/kg, respectively (dominating γ-HBCD). Finally, the authors estimated that toddlers had a higher exposure than adults, for all pollutants that were examined in the study [26].

### 2.5. Organophosphate Flame Retardants (OPFRs)

A further study by Tokumura et al. focused on the determination of organophosphate flame retardants (OPFRs) in the dust and indoor air of 25 cars. Most OPFRs were neither identified nor found in the in-cabin air of the cars at a level lower than the method quantification limit. The maximal concentration (1500 ng/m³) was found for tris(1-chloro-2-propyl) phosphate (TCIPP). Contrary to expectations, many OPFRs were identified in the dust samples gathered from the car-interior. Tris(2-ethylhexyl) phosphate (TEHP) and TCIPP were performed at the largest levels at 390 µg/g (in dust from car seats) and 640 µg/g (in dust from car floor mats), respectively. The biggest median levels (35 µg/g for car seats and 53 µg/g for car floor mats) were acquired for tris(2-butoxyethyl) phosphate (TBOEP). The characteristic hazards to OPFRs by inhalation in car compartments varied from 9.0 × 10^−4^ to 7.8 × 10^−1^ ng kg-bw(-1) day(-1) and for dust ingestion it varied from 9.2 × 10^−4^ to 8.8 × 10^−1^ ng kg-bw(-1) day(-1). By comparing these values with the reference doses for OPFRs risk to OPFRs in car cabins from dust ingestion and inhalation, it could be concluded that they were unlikely to be injurious to health [27].

OPFRs were also examined under indoor (building material markets, cars, schools, homes, offices, floor/carpet stores, and day care centers) and outdoor conditions. In indoor samples, the total OPFR levels varied from 3.30 to 751.0 ng/m^3^ (median 40.23 ng/m^3^), in contrast to the median of 5.38 ng/m^3^ in outdoor air. Tributyl phosphate (TnBP), Tris(2-chloroisopropyl)phosphate (TCPP), and tris(isobutyl)phosphate (TiBP) were commanded in outdoor as well as indoor air. Highest OPFR-concentrations were observed in cars (median 180.3 ng/m^3^), followed by schools (median 36.23 ng/m^3^), day care centers (median 31.80 ng/m^3^), building material markets (median 31.17 ng/m^3^), and homes (median 12.51 ng/m^3^), *p* < 0.05. Some studied concluded that there are 3 specific clusters of OPFRs (TCPP, TiBP/TnBP, and TEP/TCEP/TDCPP), whose accumulations are correlated with the dispersion of components prevailing in the indoor surroundings. Moreover, environmental settings (e.g., ventilation or cleaning) can influence the OPFR values [28].

An analysis of the general human exposure to organophosphate compounds showed that BDCIPP concentrations have increased dramatically. 2014/2015 BDCIPP concentrations were more than 15 times higher than in samples of 2002/2003 (10β = 16.5; 95% confidence interval from 9.64 to 28.3). Additionally, the exposure varies seasonally (higher concentrations in summer for TDCIPP and TPHP) [29].

A Greek study group found that in the interior of 25 different cars, concentrations of Σ8PFRs varied from 2000 to 190,000 ng/g (mean—20,000 ng/g, median: 11,500 ng/g), and that of Σ4ePFRs varied from 44 to 8700 ng/g (mean—1100 ng/g, median: 190 ng/g). However, finally, the intake was significantly lower than the reference doses [30].

### 2.6. Conventional Cigarettes and New Electronic Smoking Devices

A recently published study of Schober et al. showed that in-cabin smoking is a relevant topic because levels of potentially harmful items could be predicted to be high in such limited spaces. Therefore a comprehensive appreciation of dirtiness in seven passenger cars was assessed, while new electronic smoking products (IQOS, e-cigarette) and tobacco cigarettes were being smoked. For this purpose, the data were collected on the indoor air pollution and indoor climate with volatile organic compounds, fine, and ultrafine particles, while the cars were in motion. The authors found out that smoking of an IQOS had nearly no influence on the average number concentration (NC) of fine particles (>300 nm) or on the PM2.5 level in the interior. On the contrary, the NC of particles with a diameter of 25–300 nm, conspicuously rose in all cars (1.6–12.3 × 10^4^/cm³). Five of the 7 tested cars had a strong increase in the PM2.5 concentration to 75–490 µg/m³, when an e-cigarette was evaporated in the interior. The highest PM2.5 values (64–1988 µg/m³) were determined while smoking tobacco cigarettes. The level of propylene glycol increased in 5 car interiors to 50–762 µg/m³ with the e-cigarette (German indoor health precaution guide value for propylene glycol was surpassed in 3, and the health hazard guide value in 1 of them). Beyond this, in 4 cars, the nicotine value also rose to 4–10 µg/m³ while steaming the e-cigarette. The nicotine levels associated with the e-cigarette and the IQOS were assimilable, whereas the largest nicotine concentrations (8–140 µg/m³) demonstrated tobacco cigarettes. Moreover, the investigators detected that using cigarettes also induced pollution in the room atmosphere through acetaldehyde (26.5–141.5 µg/m³), formaldehyde (18.5–56.5 µg/m³), and acetone (27.8–75.8 µg/m³) [31].

Second-hand smoke exposure can often occur in the family car. An interesting review about determinations of second-hand smoke exposure in-car due to cigarette flaming showed the following results—the PM2.5 levels with at least 1 opened window differed from 47 µg/m^3^ to 12,150 µg/m^3^. With all windows closed, PM2.5 varied from 203.6 µg/m^3^ to 13,150 µg/m^3^. Even with an increased airflow from open windows or air-conditioning, smoking in cars results in a very high hazard through second-hand smoke and elevated values of atmospheric (PM2.5) and biological (cotinine, 3-hydroxycotinine, nitrosamine) parameters of exposure. 

The only possible protection against second-hand smoke within cars is not to smoke tobacco [32]. Tobacco cigarettes, the IQOS, and e-cigarettes are all preventable origins of indoor contaminants [31].

### 2.7. High Molecular Weight Plasticizer

Another relevant study of Perez et al. shows a risk and exposure evaluation of diundecyl phthalate (DUP), a high molecular weight phthalate plasticizer existing in car interiors. For this case, total daily amount of DUP was determined by measuring DUP in wipe samples from vehicle seats of 6 cars. Four of them offered divergent visible surface residue on the seats. As a comparison, 2 cars with no visible surface residue were sampled. From all seats, DUP was the prevailing organic conglomerate detected in each cleaning tissue. Furthermore, an exposure appraisal of DUP via dermal, oral, and inhalation transmission through car seats was investigated. The mean, standard deviation, and maximum DUP levels on the seats with visible surface residue were 6983+/-7823 µg/100 cm² and 38,300 µg/100 cm², respectively. The mean and 95th percentile of the mean for a daily cumulative dose of DUP for all hazard tracks for the seats, with no visible surface residue, reached values of 7 × 10^−4^ to 4 × 10^−3^ mg/kg per day and from 8 × 10^−4^ to 5 × 10^−3^ mg/kg per day, respectively. Summative measurements for seats with visible surface residue showed results from 2 × 10^−3^ to 2 × 10^−2^ mg/kg per day and from 4 × 10^−3^ to 2 × 10^−2^ mg/kg per day, respectively. The estimated daily assimilation (absorbed or contact dose) of DUP from car seats were far lower than the NOAELs (no observed adverse effect level) indicated in and derived from animal tests. Therefore, it was also well below the reported Registration, Evaluation, Authorisation, and Restriction of Chemicals (REACH) Derived No Effect Levels (DNELs) for the general population. According to the investigators, the vulnerability to DUP through car seat covers did not represent a determinable heightened health-risk [33]. 

### 2.8. Nitrogen Oxides (NOx)

In addition to the above-mentioned potential pollutants, nitrogen dioxide also needs to be considered as one of the primary pollutants from motor vehicles. A Polish study investigated in-vehicle NO_2_ levels by means of the spectrophotometric technique. The median concentrations of NO_2_ concentrations differed from 22.6 g/m^3^ to 107.7 mg/m^3^. The highest ones were noted inside cars moving on urban roads with NO_2_ levels between 97.4 mg/m^3^ and 107.7 mg/m^3^ and the lowest ones were detected near the pedestrian routes (22.6 mg/m^3^ to 47.5 mg/m^3^. The maximum NO_2_ value measured in a car travelling in city traffic was 183.2 mg/m^3^. The minimum NO_2_ value of 9.3 mg/m^3^ was found in a road in next to a residential area (low traffic intensity, pollution dispersion) [34]. 

Furthermore, a Korean study determined mean NO_2_ indoor (24.7 ± 10.7 ppb) and outdoor (23.3±8.3 ppb) levels inside and outside a cabin (mean indoor to outdoor NO_2_ ratio was 1.1). Mean individual NO_2_ exposure was 30.3 ± 9.7 ppb. There was a higher correlation between personal NO_2_ exposures and in-cabin NO_2_ concentrations (r = 0.89) than with the indoor residential NO_2_ values (r = 0.74) or with the outdoor ones (r = 0.71). Additionally, the NO_2_ exposures in LPG-fueled cars (26.3 ± 1.3 ppb) were significantly lower than those (38.1 ± 1.3 ppb) in diesel-fueled cars (*p* < 0.01) [35].

**Figure 1:**
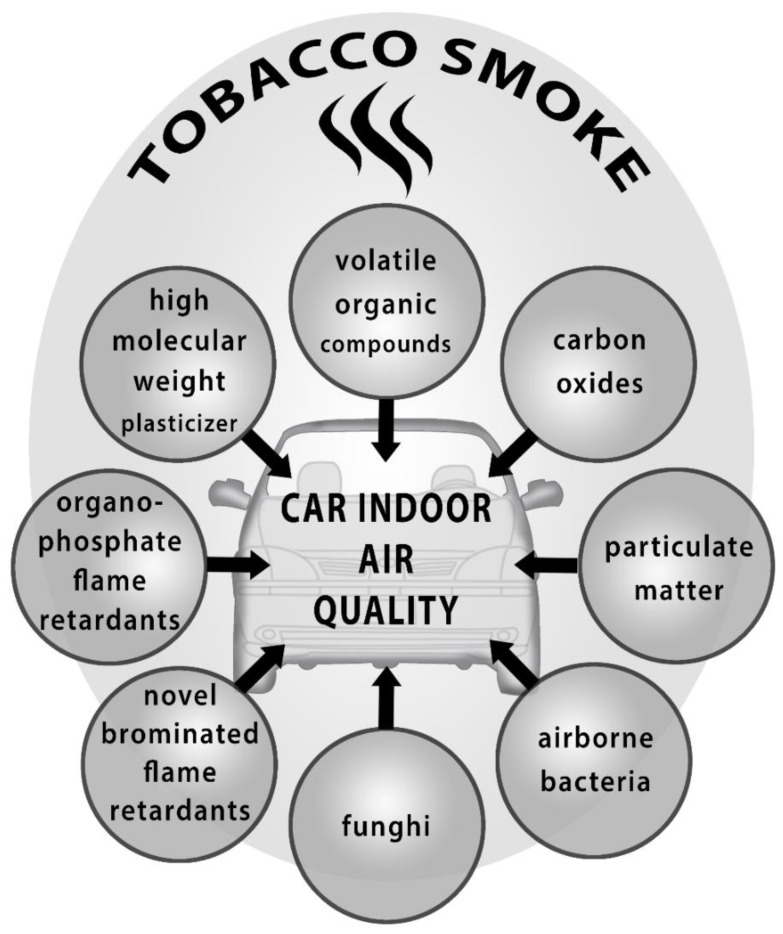
Summarized negative factors influencing a car’s indoor air quality.

Figure 1 represents the above-mentioned negative influences on indoor car-air quality. 

## 3. Conclusions

Air quality aspects influence numerous areas of environmental and occupational medicine [36,37]. Especially in today’s fast-moving and mobile age with associated lengthy periods of stay in enclosed cars, maintaining a healthy air hygiene in the car-interior is important and has attracted increasing attention.

Depending on numerous external factors, window-opening, correct usage of automated air conditioning systems, and indoor air filters could be useful air quality improvement tools and recommendations for optimizing the interior air hygiene [21]. Regarding numerous potentially deleterious indoor air hazards, there is still comparably limited data available in contrast to the multitude of data that is available for outdoor air pollution. 

Additionally, another problem is the difficulty in generalizing the influences of negligible differences in car age, condition, and manufacturer. Therefore, for more representativeness and validation, it is indispensable to strengthen the test series with more vehicles with different endowment, age, model, conditions, and so on.

So further research on this field is needed. Especially against the background of current political issues, the small amount of data on nitrogen oxides are striking. Further studies should be devoted to this topic.

In addition to this, for a better representation, further tests should be carried out in different scenarios (variation within the car, speeds, air conditioning, ventilation, window-opening, sunroof-opening, measurement in the car, number of passengers, etc.) under standardized laboratory conditions. Henceforward, mechanistic models could better analyze the in-vehicle pollution and exposure.

Generally, the results should lead to changes in the field of urban road transport by political members [34]. From a medical point of view, further studies are needed to determine if exposure to these levels of pollutants in the general population are related to adverse health effects.
